# COVID arm as a common cutaneous manifestation after mRNA-1273 vaccination: a systematic review

**DOI:** 10.1186/s12879-022-07973-4

**Published:** 2023-01-06

**Authors:** Maulidina Agustin, Monica Trifitriana, Retno Danarti

**Affiliations:** 1 Faculty of Medicine, Public Health, and Nursing, Universitas Gadjah Mada, Yogyakarta, Indonesia; 2 Faculty of Medicine, Universitas Sriwijaya, Palembang, Indonesia; 3 Department of Dermatology and Venereology, Faculty of Medicine, Public Health, and Nursing, Universitas Gadjah Mada, Gedung Radiopoetro Lantai 3, Jalan Farmako, Sekip, Yogyakarta, 55281 Indonesia

**Keywords:** SARS-CoV-2, COVID arm, Cutaneous manifestation, Skin rash, mRNA-1273, Vaccination

## Abstract

**Background:**

By August 2022, CoronaVirus Disease-2019 (COVID-19) had caused 600 million illnesses and 6.5 million fatalities globally. A massive vaccination program is being implemented worldwide to suppress this condition. Several works of literature stated that mRNA COVID-19 vaccination, specifically with the mRNA-1273 vaccine, is followed by clear evidence of the COVID arm effects associated with this vaccine.

**Objective:**

To analyze the latest evidence of COVID arm as a common effect of mRNA-1273 vaccination with the ultimate goal of improving vaccine counseling to help healthcare professionals and reassure patients.

**Methods:**

A comprehensive search was performed on topics that assess the COVID arm as a cutaneous manifestation following mRNA-1273 vaccination from inception up until July 2022.

**Results:**

Eighteen studies with a total of 1129 participants after the first and second dose of mRNA-1273 vaccination reported that most participants had COVID arm following the first dose administration. The characteristics of the patients were a mean age of 43.8 years old, and females represented ≥ 50% in most studies, with a mean onset of 6.9 days after the first dose administration. Symptoms resolved within seven days following the treatment and were harmless.

**Conclusions:**

This study found that the COVID arm condition is most common following the first mRNA-1273 vaccination in the female and middle-aged group. The correlation between demographic variables and COVID arm risk elucidates that the reaction is a type IV allergic skin reaction.

## Introduction

The Severe Acute Respiratory Syndrome Coronavirus 2 (SARS-CoV-2) infection has spread widely and rapidly to become the globally hazardous and challenging Coronavirus Disease 2019 (COVID-19) pandemic. This highly infectious disease has negatively impacted the state of health and economics of the world [1]. By August 2022, COVID-19 had caused 600 million illnesses and 6.5 million fatalities globally [2]. A massive vaccination program is being implemented worldwide to suppress the COVID-19 pandemic [3]. Despite being praised as a scientific breakthrough, the rapid production of two SARS-CoV-2 viral messenger-RNA (mRNA) vaccines approved by the United States Food and Drug Administration (US-FDA) has caused worries about unfavorable allergic reactions [4].

The US FDA granted Emergency Use Authorizations for two mRNA COVID-19 vaccines in December 2020. These vaccines were produced by Pfizer-BioNTech (Pfizer Inc., New York, New York, and BioNTech SE, Mainz, Germany), Moderna (Moderna Inc., Cambridge, Massachusetts), and more than 300 million doses have been given in the United States [5]. Clinical trials for both vaccines reported local injection site reactions and systemic symptoms after both doses [6]. Although immediate hypersensitivity to vaccinations has received much attention, delayed reactions, such as delayed cutaneous eruptions, can occur and have been observed in clinical studies [7]. In this study, analyzing the skin reactions of COVID-19 vaccination data, in particular, may help us learn more and give helpful information to define the changes in cutaneous reactions and timing of cutaneous reactions to the mRNA-1273 vaccines to improve vaccine counseling.

## Methods

### Search strategy

**Fig. 1:**
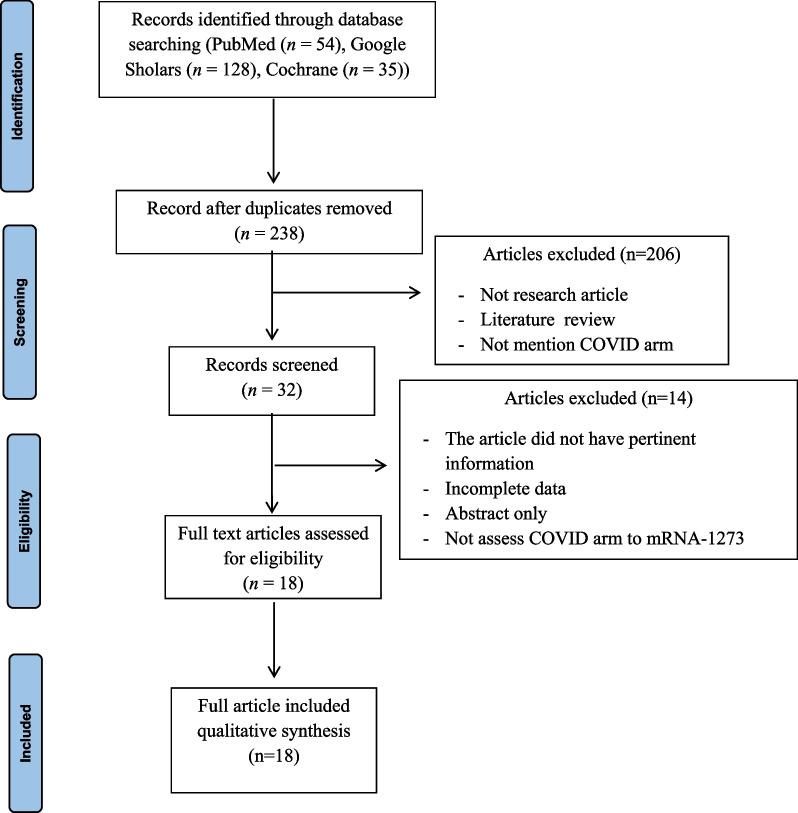
Prisma Flow Diagram

The literature search was completed in July 2022 from three databases, which were PubMed, Google Scholar, and Cochrane. The keywords used were ("COVID-19" OR "SARS-CoV-2") AND ("Moderna" OR “mRNA-1273”) AND (“Skin reactions” OR “Skin manifestation” OR “Cutaneous reactions” OR “Cutaneous manifestation” OR “Skin rash”). The records were then systematically evaluated based on the inclusion and exclusion criteria. Two authors (MA, MT) independently performed an initial search (scanned all abstracts to find the relevant studies). When discrepancies occurred, the third author (RD) made the final determination, using similar procedures for any potential discrepancies described above, to determine the suitability of the full-text article. All the chosen articles were re-read by three authors independently. Figure 1 shows the Preferred Reporting Elements for Systematic Reviews and Meta-Analyses (PRISMA) [8] flowcharts for a research literature search strategy.

### Selection criteria

The inclusion criteria for this study are all studies that assess COVID arm as an effect of mRNA-1273 vaccination. Study designs from the selected publications included case reports, case series, prospective and retrospective cohort studies, case–control studies, and clinical trials. The exclusion criteria for this study are studies that did not report the COVID arm effect of mRNA-1273 vaccination, reactions after booster vaccination because the number was minimal (insufficient data), and study designs, including review articles, meta-analyses, and editorials. Figure 1 provides a summary of the study selection.

### Data extraction and quality assessment

Data extraction and quality assessment were performed by three independent authors (MA, MT, and RD). After removing duplicate articles, chosen studies were included to be analyzed. Data collected included authors, study design, age, gender, history of the disease, the cutaneous manifestation onset after the first and second dose vaccination, histopathology finding, treatment, and outcome. Any disagreements at the time were reconciled by discussion. The methodological quality of each included study was assessed using Robin-I analysis. This scale has several criteria, as shown in Table 1. Eighteen included studies were generally of high quality. The risk of bias was low for all included studies.

**Table 1:** ROBINS-I analysis

Study	Confounding bias	participants bias	intervention bias	Missing data bias	Outcome bias	Reporting bias	Overall risk bias
Blumenthal et al. [19]	No	No	No	No	No	No	Low
Catala et al. [24]	No	No	No	Yes	No	No	Low
Guerrero et al. [25]	No	No	No	No	No	No	Low
Gregoriou et al. [26]	No	No	No	No	No	No	Low
Higashino et al. [9]	No	Yes	No	No	No	No	Low
Hoff et al. [11]	No	No	No	No	No	No	Low
Jacobson et al. [14]	No	No	No	No	No	No	Low
Johnston et al. [27]	No	No	No	No	No	No	Low
Larson et al. [4]	No	No	No	No	No	No	Low
Lindgren et al. [16]	No	No	No	No	No	No	Low
McMahon et al. [6]	No	No	No	No	No	No	Low
Papamanoli et al. [28]	No	No	No	No	No	No	Low
Picone et al. [29]	No	No	No	No	No	No	Low
Robb et al. [30]	No	No	No	No	No	No	Low
Tihy et al. [31]	No	No	No	No	No	No	Low
Wei et al. [32]	No	No	No	No	No	No	Low
Xu et al. [33]	No	No	No	No	No	No	Low
Zengarini et al. [34]	No	No	No	No	No	No	Low

Bias analysis shows that all journals have a clear population, intervention, comparison, and outcome. These journals are mostly case series, followed by cross-sectional and cohort retrospectives. There is 1 journal with missing data bias and 1 journal with participant bias

### Definition

The term “COVID arm” in the US or “Moderna arm” in Japan is a local, delayed-onset, transient, adverse cutaneous manifestation around the vaccination injection site [9] Local site reactions were considered as occurring within three days of the initial dose of vaccination, whereas delayed large local reactions occurred four or more days later [6]. COVID arm is characterized by localized erythema, swelling, rash, and/or induration, pain, burning, and pruritus [6, 10].

## Results

### Baseline characteristics of included studies

We summarize the results of eighteen studies (Table 2) with 1129 COVID arm reactions following mRNA-1273 (Moderna) vaccination two were cross-sectional studies, two were cohort retrospective studies, nine were case series, three were case reports, and one was a registry-based study. As shown in Table 3, eighteen studies involving 1129 patients with a weighted average of the mean age of the COVID arm was 43.8 years old. Overall, females represented ≥ 50% of cases in most studies with a total number of 745 (65.9%) participants. Only two studies reported the same prevalence between males and females.

**Table 2:** Characteristics of included studies

No	Study	Study design	Age, mean (SD)	Gender (female/male)	History disease	After the first dose				After the second dose			
						Onset, mean(SD)	Histopathology finding	Treatment	Resolution, mean(SD)	Onset, mean (SD)	Histopathology finding	Treatment	Outcome (mean (SD))
1	Blumenthal et al. [19]	Case series	43.3 (8.6)	10/2	Allergy history (contrast, rhinitis, penicillin, urticaria, wasp, almond, drugs)	8.3 d (1.6)	N/A	Topical corticosteroids, oral antihistamines, analgetics, antibiotics	14.4 d (2.1)	N/A	N/A	N/A	N/A
2	Catala et al. [24]	Crossectional	N/A	91 (total)	N/A	4.9 d (3.7)	N/A	N/A	7.4 d (4.1)	N/A	N/A	N/A	N/A
3	Guerrero et al. [25]	Case series	44.1 (10,4)	13/0	Allergy history (rhinoconjunctivitis, asthma, chronic urticaria, latex anaphylaxis)	4.5 d (3.2)	N/A	Topical corticosteroid, oral antihistamin	4,3 d (1,7)	1.1 d (1.1)	N/A	None	1.6 d (1.4)
4	Gregoriou et al. [26]	Case series	59 (12.1)	3/0	N/A	9 d (0)	N/A	Topical corticosteroid, oral antihistamine	4 d (1)	N/A	N/A	N/A	N/A
5	Higashino et al. [9]	Crossectional	43,5 (36,06)	577/170	N/A	7.24 d (1.41)	N/A	N/A	5.72 d (4.22)	N/A	N/A	N/A	N/A
6	Hoff et al. [11]	Case series	50 (13.8)	9/2	Obesity	7.1 d (2.8)	N/A	Oral antihistamines, topical glucocorticoids	2.6 d (1.2)	3 d (1.0)	Superficial and deep perivascular inflammatory infiltrate in the dermis The perivascular infiltrate was dominated by lymphocytes	Oral antihistamines, topical glucocorticoids	1.6 d (0.6)
7	Jacobson et al. [14]	Cohort retrospective	40.2 (7.8)	14/0	Atopic/allergic history	6.2 d (2.2)	Superficial and deep lymphohistiocytic infiltrate with scattered admixed interstitial neutrophils and eosinophils	N/A	4.2 d (1.9)	2.1 d (0.4)	N/A	N/A	4.3 d (1.9)
8	Johnston et al. [27]	Case series	38 (median)	13/3	Atopic allergic	6.3 d (3.1)	N/A	Topical clobetasol, hydrocortisone, cephalexin, and antihistamine	6.3 d (5.7)	1.4 d (1.3)	N/A	Topical clobetasol, hydrocortisone, and antihistamin	2.3 d (1.8)
9	Larson et al. [4]	Case series	71	1/0	None	7 d	Superficial and mid-perivascular infiltrate comprised of lymphocytes and eosinophils with focal vacuolar at the dermal–epidermal junction	N/A	N/A	N/A	N/A	N/A	N/A
10	Lindgren et al. [16]	Case series	46.5 (19.1)	2/0	N/A	6.5 (0.7)	N/A	Topical corticosteroid, antibiotics	2.5 (2.1)	N/A	N/A	N/A	N/A
11	McMahon et al. [6]	Registry-based study	45 (median)	206 (total), 93% F	N/A	7 d (median)	N/A	Topical corticosteroids, oral antihistamines, analgetics, ice, antibiotics	4 d (median)	2 d (median)	N/A	Topical corticosteroids, oral antihistamines, analgetics, ice, antibiotics	3 d (median)
12	Papamanoli et al. [28]	Case report	N/A	1/1	None	9 d (2.8)	N/A	Antibiotics cephalexin	3.5 d (0.7)	N/A	N/A	N/A	N/A
13	Picone et al. [29]	Case series	61 (1.4)	1/1	N/A	7 d	N/A	Topical corticosteroids, emollients	± 14 d	N/A	N/A	N/A	N/A
14	Robb et al. [30]	Case report	74	1/0	N/A	8 d	N/A	Cold compress	5 d	N/A	N/A	N/A	N/A
15	Tihy et al. [31]	Case series	38	1/0	N/A	N/A	N/A	N/A	N/A	5 d	Dilated capillaries and venules in the superficial and mid dermis, lymphocytic perivascular infiltrate with rare neutrophils and eosinophils	Topical corticosteroids, oral antihistamines, analgetics, antibiotics	< 2 wks
16	Wei et al. [32]	Case series	65.5 (9.2)	4/0	Psoriasis, atrial fibrillation, hypothyroidism	8.2 d (1.2)	N/A	Topical clobetasol, mometasone furoate 0.01%, cephalexin and antihistamin	4 d (2.1)	N/A	N/A	N/A	N/A
17	Xu et al. [33]	Cohort retrospective	44.5 (2.1)	2/0	Allergy history	3.5 d (0.7)	N/A	Antihistamine. H2 blocker, oral steroid	1 d	N/A	N/A	N/A	N/A
18	Zengarini et al. [34]	Case report	63	1/0	None	5 d	N/A	Cold compress	2 d	N/A	N/A	N/A	N/A

**Table 3:** Demographic characteristics of COVID arm following mRNA-1273 Moderna vaccine

Characteristic	Participants N.o (%)
Total	1129 (100)
Sex^a^	
Female	745 (65.9)
Male	348 (34.1)
Patient age, mean^a^	43.8
Past dermatologic history	
None	12 (1.0)
Psoriasis	1 (0.0)
Atopic dermatitis	2 (0.1)
Urticaria	6 (0.5)
Unknown	1111 (98.4)
Past medical history	
None	24 (2.1)
Atopic allergy	30 (2.6)
Atrial fibrillation	1 (0.0)
Hypothyroidism	1 (0.0)
Morbid obesity	1 (0.0)
Unknown	1074 (95.1)
Medication	
None	41 (3.6)
Topical corticosteroid	23 (2.0)
Emollients	2 (0.1)
Oral corticosteroid	3 (0.2)
Oral antihistamine	23 (2.0)
NSAIDs	2 (0.1)
Antibiotics	4 (0.3)
Unknown	1047 (92.7)
Reaction to dose	
First dose	997 (88.3)
Second dose	66 (5.8)
Recurrent	35 (3.1)

^a^Some studies not included the total numbers of participant in each group details

### Medical history

**Fig. 2:**
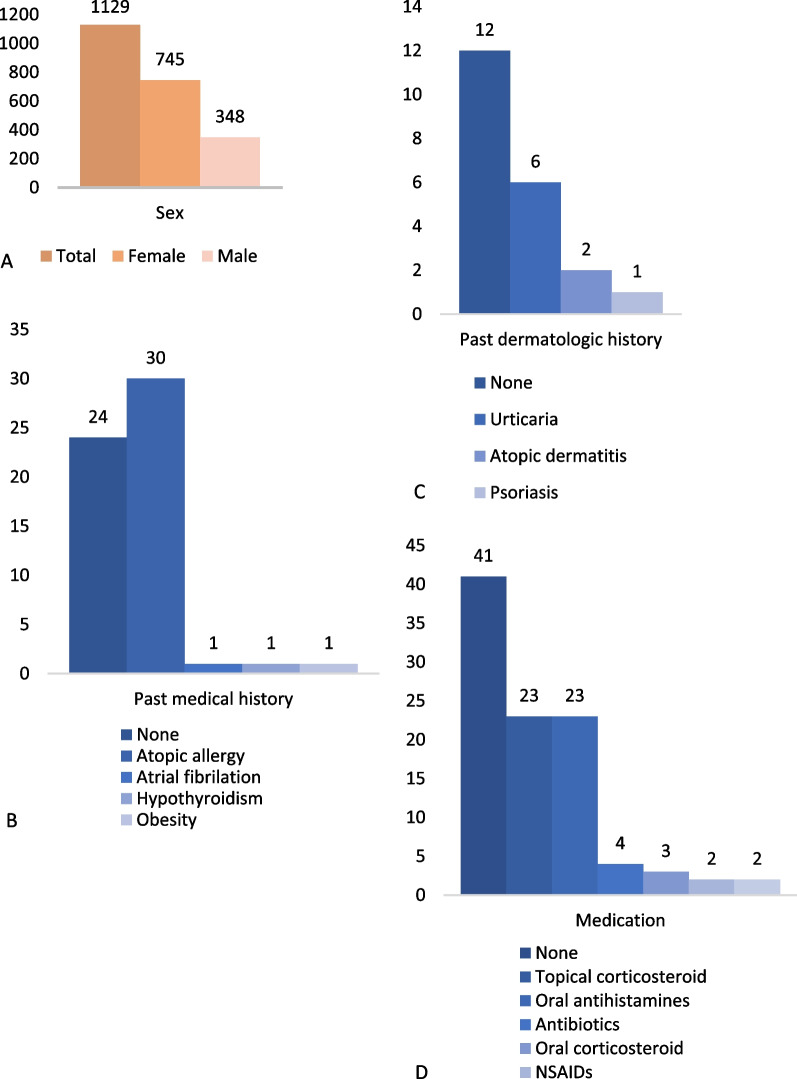
Summary findings of COVID arm patient’s characteristics following mRNA-1273 Moderna vaccine

The medical history of the patients was reported in ten studies. Atopic allergy in 30 patients (2.6%) was the most commonly reported to the previous medical history of the participants, followed by 24 patients (2.1%) without preexisting comorbidities. While other reported medical histories were atrial fibrillation, hypothyroidism, and obesity (Fig. 2). However, 12 patients (1%) were reported without any previous dermatological history followed by urticaria findings in 6 patients (0.5%).

### Clinical course and prognosis

The highest incidence of COVID arm cases occurred after the first dose of mRNA-1273 vaccination in 997(88.3%) cases, with a mean onset of 6.9 days following the first dose administration. Among the reports included, the mean of symptom resolution was 5 days following the treatment. On the other hand, the second-dose COVID arm incidence was fewer in number found in 66 (5.8%) cases and occurred more quickly than the first-dose COVID arm cases. Histopathology findings from studies found superficial perivascular inflammatory infiltrate in the dermis with a dominance of lymphocytes. Most of COVID arm cases were not required medical treatment, followed by topical corticosteroids were used in 23 (2%) cases [6, 11, 16, 19, 24–27, 29, 32] and oral antihistamines in 23 (2%) cases [6, 11, 19, 25–27, 31, 32] were used. In contrast, others reported the administration of oral corticosteroids, antibiotics, and NSAIDs as a pain relievers [6, 16, 19, 24, 27, 28, 31–33].

## Discussion

A registry-based study by Baden et al*.* [7] that included 30,420 participants in the United States revealed that delayed injection-site reactions following COVID-19 vaccination were found in 244 recipients (0.8%) after the first dose and in 68 recipients (0.2%) after the second dose. A recent study in Germany reporting delayed reaction symptoms after mRNA-1273 vaccination showed an incidence rate of 1.1% in female recipients and 0.16% in the general population [11]. Similar results were observed in a Japanese study (2021) which found that reactions were more frequent in the female population (12.5%) [12]. Our study revealed that COVID arm was commonly found in the female population, even though females are known to have higher reactogenicity to immunizations, which may imply an actual difference or reporting bias [13]. Other hypotheses, such as weight loss, sex differences in pharmacokinetics and pharmacodynamics, and sex differences in health information retrieval behavior have been proposed [14, 15].

COVID arm as a delayed allergic reaction is seen as localized, self-resolving occurrences that do not develop systemically and hence do not exclude future immunizations [16, 17]. Many vaccine components, including lipid nanoparticles in mRNA vaccines, PEGs, polysorbates, dimyristoyl glycerol, thimerosal, and tromethamine, can behave as haptens [18]. The active components in the mRNA-1273 vaccines are mRNA and lipids. Though PEG2000 and polysorbate 80 are the only chemicals in this vaccination that have previously been found to produce delayed hypersensitivity responses, more study is needed to discover if these compounds are the causes of COVID arm [19, 20]. Reactivation of particular memory T cells in previously sensitized individuals soon results in an influx of diverse inflammatory cells, including Th1 cells. As a result, acute spongiotic dermatitis develops beyond the scope of a "typical" injection site response [21]. The phase 3 clinical trial of the mRNA-1273 vaccine revealed delayed injection-site reactions occurred in 244 of the 30,420 participants (0.8%) after the first dose and 68 individuals (0.2%) after the second dose. These findings are highly suspected of delayed-type hypersensitivity reactions (DTR). Usually, the DTR, which is mediated by macrophage and T cell interactions and is supported by histopathology, showed that predominantly lymphocyte as an effect of T cell-mediated after mRNA-1273 vaccination [21, 22].

The most commonly reported cutaneous finding was local reaction findings with a mean onset of 6.9 days after the first vaccine administration. While the second-dose cutaneous reactions onset occurred more quickly (day 1–2) and were generally lesser, which suggests sensitized individuals tolerated the vaccine better [23]. Most of COVID-arm patients resolved spontaneously, several studies proved the use of cold compress is beneficial [6, 30, 34]. This study showed that patients responded well to topical corticosteroids, oral antihistamines, and pain relievers. and some others were self-limiting. These reactions resolved after three days. Antibiotics were required several studies, in Blumenthal et al*.* [19] study oral antibiotic with concern that the lesion might be cellulitis.

In this study, we found COVID arm manifestation following mRNA-1273 vaccination occurred more frequently among individuals with mean age older than 40 years old. The histopathology in several studies revealed spongiosis of the epidermis with scattered admixed interstitial neutrophils and eosinophils. These findings are similar to contact dermatitis, which suggests COVID arm as a type IV allergic skin reaction.

## Limitation

This study primarily consists of case series. Further research is needed to characterize the epidemiology, compare the incidence or severity of cutaneous reaction by vaccine type, and classify the histopathology findings.

## Conclusion

This study found that the COVID arm is most common following the first dose of mRNA-1273 vaccination in females and the middle-aged group (40–65 years old). The correlation between demographic variables and COVID arm risk elucidates that the reaction is a type IV allergic skin reaction. Further large-scale studies are warranted to identify the COVID arm effect after mRNA-1273 vaccination with more detail and verification.

## Data Availability

The datasets collected and analyzed within this study are available from the corresponding author on reasonable request.
